# Computational models with thermodynamic and composition features improve siRNA design

**DOI:** 10.1186/1471-2105-7-65

**Published:** 2006-02-12

**Authors:** Svetlana A Shabalina, Alexey N Spiridonov, Aleksey Y Ogurtsov

**Affiliations:** 1 National Center for Biotechnology Information, National Library of Medicine, National Institute of Health, Bethesda, MD 20894, USA; 2 Department of Applied Mathematics, Massachusetts Institute of Technology, Cambridge, MA 02139, USA

## Abstract

**Background:**

Small interfering RNAs (siRNAs) have become an important tool in cell and molecular biology. Reliable design of siRNA molecules is essential for the needs of large functional genomics projects.

**Results:**

To improve the design of efficient siRNA molecules, we performed a comparative, thermodynamic and correlation analysis on a heterogeneous set of 653 siRNAs collected from the literature. We used this training set to select siRNA features and optimize computational models. We identified 18 parameters that correlate significantly with silencing efficiency. Some of these parameters characterize only the siRNA sequence, while others involve the whole mRNA. Most importantly, we derived an siRNA position-dependent consensus, and optimized the free-energy difference of the 5' and 3' terminal dinucleotides of the siRNA antisense strand. The position-dependent consensus is based on correlation and *t*-test analyses of the training set, and accounts for both significantly preferred and avoided nucleotides in all sequence positions. On the training set, the two parameters' correlation with silencing efficiency was 0.5 and 0.36, respectively. Among other features, a dinucleotide content index and the frequency of potential targets for siRNA in the mRNA added predictive power to our model (*R *= 0.55). We showed that our model is effective for predicting the efficiency of siRNAs at different concentrations.

We optimized a neural network model on our training set using three parameters characterizing the siRNA sequence, and predicted efficiencies for the test siRNA dataset recently published by Novartis. On this validation set, the correlation coefficient between predicted and observed efficiency was 0.75. Using the same model, we performed a transcriptome-wide analysis of optimal siRNA targets for 22,600 human mRNAs.

**Conclusion:**

We demonstrated that the properties of the siRNAs themselves are essential for efficient RNA interference. The 5' ends of antisense strands of efficient siRNAs are U-rich and possess a content similarity to the pyrimidine-rich oligonucleotides interacting with the polypurine RNA tracks that are recognized by RNase H. The advantage of our method over similar methods is the small number of parameters. As a result, our method requires a much smaller training set to produce consistent results. Other mRNA features, though expensive to compute, can slightly improve our model.

## Background

RNA interference (RNAi) is a biologically important mechanism, and a medically promising method of suppressing gene expression in eukaryotic cells. Two main types of interfering RNAs (endogenous microRNAs, and exogenous short interfering RNAs) silence genes by complementary interactions with their mRNA targets. Both microRNAs (miRNAs) and small interfering RNAs (siRNAs) exhibit strand bias and are incorporated into related RNA-induced silencing complexes (RISCs). The assembly of the RISC is a key step in RNA interference. Enhanced flexibility of miRNA precursors at the 5'-antisense terminal base pair was demonstrated, and the same trend was observed in siRNAs [1]. siRNA duplexes are functionally asymmetric: one of the two strands is preferable for incorporation to the RISC. Tomari and coauthors [2] showed that thermodynamic differences in the base-pairing stabilities of the 5' ends of the two siRNA strands determine which strand is assembled into the RISC. Stability of the base pairs at the 5' ends of the two siRNA strands determine the degree to which each strand participates in the RNAi pathway [3].

Recent studies suggest that siRNAs and miRNAs are functionally interchangeable, with the choice of mRNA translational repression versus mRNA cleavage determined solely by the degree of complementarity between small RNAs and their targets [4-6]. Both siRNA strands of the siRNA duplex can direct RNAi, because each RISC contains only one of the two strands of the siRNA duplex [7-10]. In a recent study, Hu and co-authors [11] evaluated the relative gene-silencing efficiency of a pair of sense and antisense transcripts, derived from the same genetic locus but transcribed in opposite directions. The study demonstrated that siRNAs can induce degradation of a sense and an antisense transcripts simultaneously and that the strand-specific activity of siRNA can be altered by single-base mismatches [11]. Previous studies also showed that the thermodynamic profiles of siRNAs and miRNAs and asymmetry in these profiles play a key role in RNAi efficiency [12].

A number of siRNA-specific features contributing to the different steps of the RNA-mediated gene silencing were identified and several sets of prediction rules were suggested and experimentally validated [8-14]. Ui-Tei et al. [15] showed, using a set of ~100 siRNAs, that highly effective siRNAs satisfy the following criteria: A/U at the 5' end and AU-richness in the 5' terminal 7 bp region of the antisense strand, G/C at the 5' end of the sense strand, and the absence of any long GC stretch of more than 9 bp in length. Composition features for functional miRNAs were studied by Krol et al. [16]. Other authors showed that the secondary structure of mRNAs can influence the efficiency of siRNAs [17-20]. Khvorova et al. [1] and Schwarz et al. [3] accented on the thermodynamic features of siRNA duplexes. Later Stockholm's rules were suggested, where a combination of thermodynamic and statistical features was used [21]. Amarzguioui and Prydz [22] found that some features consistently correlated with functionality, these include an asymmetry in the stability of the duplex ends and their nucleotide composition preferences. Saetrom and Snove [23] compared different prediction methods and rules and showed that the best models gave an overall correlation between predicted and observed efficacy of 0.46 on siRNA (data for 101 efficient and 103 inefficient siRNAs). Recently, the algorithm BIOPREDsi was used to predict the activities of 249 siRNAs from the 2431 siRNA set by Novartis, targeting 34 mRNAs (*R *= 0.66). This algorithm was trained on the remaining 2,182 siRNAs [24]. However, this training set may have contained siRNAs partially overlapping with the test set. According to our calculations, failure to account for test/training overlaps can lead one to substantially overestimate *R *(see "Results, A note about cross-validation" in the present paper). Our training procedure does not have this problem.

In this study we used a combination of different composition and thermodynamic characteristics in a computational model. We derived the list of parameters, which differ significantly between sets of efficient and inefficient siRNAs. We also analyzed common thermodynamic and statistical features in naturally occurring miRNAs. Functional miRNAs have the same trends as efficient siRNAs for some of these features. The difference between free energy for terminal dinucleotides at both 5' and 3' ends of siRNA antisense strand has the capacity to discriminate between the antisense and sense strands of the duplex, similar to miRNAs [16]. Antisense strands of efficient siRNAs are U-rich, especially at the 5' end and resemble the pyrimidine-rich oligonucleotides interacting with the polypurine RNA tracks recognized by RNase H. These observations together with experimental evidence on the structural similarity between the family of RNase H enzymes and Piwi domain of Argonaute protein [25], suggest that there is some similarity in interactions of those proteins with nucleic acids. Our best neural network model for prediction of efficient siRNAs used three parameters and produced a correlation of 0.75 between predicted and observed efficiencies on the validation set. We also showed that our models are effective for predicting the efficiency of RNAi at low and high siRNA concentrations. The difference from previous artificial neural network applications and the novelty of our work is that our approach is based on both thermodynamic and composition features. Another advantage of our method compared with BIOPREDsi is the small number of parameters. We can use only three parameters rather than 84, so our method requires a much smaller training sets to produce consistent results. Our method can be used with smaller sets of experimental data produced under different experimental conditions.

## Results

In order to identify features possessing predictive power for efficient siRNA design, we analyzed the training set of 653 siRNAs with experimentally measured activities [see Additional file 4]. We compared commonly used parameters [26] and suggested some new ones. The known parameters we used are nucleotide contents, thermodynamic profiles of siRNA duplexes and the free energy difference between the 5' ends of the sense and antisense strands. The last one we optimized using experimental data by Schwarz et al. [3] and Hu et al. [11]. The new parameters are the dinucleotide content indexes, the summarized position-dependent consensus, the frequency of potential targets for siRNA in the mRNA, and some thermodynamic features dependent on the ΔG of inter- and intra-molecular interactions. The new parameters are described in more details in the following subsections. We selected 18 parameters that had a significant correlation with siRNA silencing activity (see Materials and Methods). Table 1 lists these parameters and their stability values calculated for four subsets. The values for 10 subsets follow essentially the same ordering, and so are omitted.

**Table 1:** Classification of the parameters for siRNA design. The stability values for the best 12 parameters are presented in bold. Four most stable parameters possessing the best predictive power are shown in bold italic.

Features	*R*	*S*_4_	*t*-test *P*
Composition features:			
***position-dependent nucleotide consensus: sum***	*0.51*	***0.047***	*2.8e-39*
position-dependent nucleotide consensus: avoided	0.44	**0.053**	9.7e-30
position-dependent nucleotide consensus: preferred	-0.446	**0.051**	1.3e-28
nucleotide content: G	0.189	0.069	6.0e-08
nucleotide content: U	-0.187	**0.065**	1.1e-05
***avoided dinucleotide content index***	*0.259*	***0.061***	*5.7e-10*
preferred dinucleotide content index	-0.212	**0.061**	5.7e-0.9
Thermodynamic features:			
ΔG of sense-antisense siRNA duplexes	-0.173	0.072	0.0003
siRNA antisense strand intra-molecular structure stability (ΔG)	-0.198	0.072	7.6e-06
stability (ΔG) of dimers of siRNAs antisense strands	-0.169	0.072	0.0001
stability profile (ΔG) for each two neighboring base pairs in the siRNA sense-antisense:			
position 1	-0.297	**0.059**	1.3e-10
position 2	-0.172	**0.065**	0.0007
position 6	-0.172	0.069	0.0004
position 13	-0.201	**0.065**	1.3e-07
position 14	-0.126	**0.065**	0.008
position 18	0.182	0.069	3.8e-06
***ΔG difference between position 1 and 18***	*0.352*	***0.055***	*7.4e-20*
local target mRNA stabilities (ΔG)	0.149	0.068	0.0008
***number of potential target copies in mRNAs (ΔG threshold)***	*-0.145*	***0.065***	*1.8e-05*

We calculated distributions of a variety of thermodynamic and composition parameters for the set of efficient and inefficient siRNAs [see Additional file 1]. Correlations among the selected parameters within the complete set of siRNAs are presented in Supplementary Materials [see Additional file 6]. Overall block scheme of the computational steps implemented in this study is presented in Supplementary Materials [see Additional file 2].

We performed a similar analysis for human and rodent miRNAs, and calculated parameters analogous to the ones we used for efficient siRNAs.

Below we discuss a number of parameters that are important for efficient siRNA design, including the features of the antisense strand of the siRNA duplex, the mRNA properties and the characteristics of the siRNA-mRNA interactions that are important for efficient siRNA design.

### Composition features

Human miRNAs and efficient siRNAs both tend to be U-rich. Efficient siRNAs are depleted in G. Significant positive and negative correlations between dinucleotide frequencies and siRNA efficacies were identified [see Additional file 4], which indicated that specific dinucleotide combinations could be helpful for optimal siRNA prediction.

We calculated context indexes for preferred and avoided dinucleotides in the subsets of highly efficient and inefficient siRNAs. The content index for preferred dinucleotides (for UC, UU) is the sum of dinucleotide counts occurring frequently in the antisense strand of siRNA efficient duplexes [see Additional file 1]. These dinucleotide counts have a significant negative correlation (*R *< -0.1) with reported activity, and so their presence indicates a more efficient siRNA. Conversely, the content index for avoided dinucleotides (for CA, GC, and GG) is the sum of the dinucleotide counts significantly correlated with reported residual target mRNA level *R *> 0.1 [see Additional file 1]. An example demonstrating how the avoided dinucleotide content index is calculated from siRNA sequence is shown in Supplementary Materials [see Additional file 3].

Frequencies of dinucleotide occurrence for miRNAs were compared to random RNA controls having the same base distribution, and the *t*-value that reflected dinucleotide bias was calculated for miRNAs. Among 207 human and 626 rodent miRNA sequences examined, the frequencies of some dinucleotides were not randomly distributed. Similar results were obtained for non-redundant set of miRNAs. miRNAs have a positive bias for oligonucleotides with alternating GC- and AU-content. Usually, A/U-containing dinucleotides (e.g. AU, UU, AA or UA) alternate with G/C-containing dinucleotides (e.g. GG, CC, GC or CG). Decreased frequencies of some G/C-containing dinucleotides are observed in both efficient siRNAs and miRNAs, as compared with the random model. G and U content correlates with siRNA efficiencies, but correlation coefficients are somewhat lower than for dinucleotide indexes.

### Position-dependent consensus

**Figure 1:**
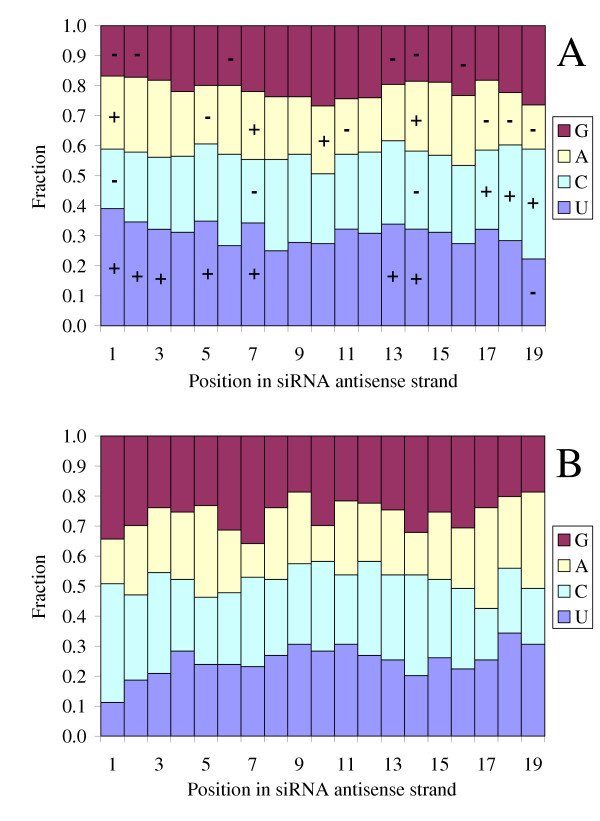
**Position-dependent consensus for efficient (A) and inefficient (B) siRNAs**. Position-dependent consensus for efficient siRNAs shows some important positions for preferable (+) or avoided (-) nucleotides.

The analysis of a large set of data allowed us to determine position-dependent nucleotide preferences and avoidances for efficient siRNAs (Figure 1). Oligonucleotide sequences were recorded in a numerical code (A->1,0,0,0; T->0,1,0,0; C->0,0,1,0; G->0,0,0,1) and correlation and t-test analyses were performed. We used a *t*-test to determine which position-nucleotide combinations were significantly (*p *< 0.03) preferred or avoided. Table 2 presents these significant *p*-values for one-tailed, two sample, unequal variance *t*-tests comparing the mean activity for siRNAs, which had the given nucleotide in the given position, with the mean activity of the remaining siRNAs. The position-dependent nucleotide preferences and avoidances are quantified by the resulting *p*-values.

**Table 2:** Determination of position-dependent nucleotide preferences and avoidances by *t*-test analysis.

Nucleotide and position		*t*-test *p*-value	Average activity if this nucleotide is:	
		
Preferred	Avoided		present	absent
U1		5.6e-13	29.78	47.49
	C1	3e-07	53.04	38.64
C19		2.9e-06	34.19	45.95
	A19	2.1e-05	51.80	39.84
	G1	8.3e-05	51.59	39.94
U13		0.00058	36.39	45.03
U2		0.00108	37.17	44.75
	G2	0.00155	49.75	40.49
	G7	0.00209	48.71	40.42
A7		0.00253	36.15	44.11
U7		0.00296	37.43	44.65
	G13	0.00433	47.84	40.78
A1		0.00523	37.31	44.04
	G6	0.00557	48.24	40.69
A10		0.00573	36.61	43.96
U14		0.00633	37.75	44.27
C17		0.00768	37.49	44.04
	C7	0.00854	47.67	40.79
A14		0.00972	37.36	43.83
	A17	0.01011	47.54	40.73
	A5	0.0104	47.31	40.85
	G14	0.01121	47.59	40.88
U3		0.01599	38.01	44.03
C18		0.01653	38.35	44.03
	C14	0.02243	46.65	40.87
	A11	0.02601	47.00	41.20
U5		0.02657	39.01	44.06
	G16	0.02902	46.56	41.09
	U19	0.02982	46.47	41.09

We combined these nucleotide biases into a preferred and an avoided position-dependent consensus indexes. The preferred position-dependent consensus index is the number of preferred nucleotides in all positions of the siRNA antisense strand. The total number of positions which have significantly preferred nucleotides is 11 in our dataset (Table 2). Thus, the preferred consensus index is an integer from 0 to 11. The avoided consensus index is defined similarly (with a maximum value of 10). The summarized position-dependent consensus is defined as "preferred minus avoided". The total number of positions involved in the summarized position-dependent consensus is 14 (*p *< 0.03).

The avoided and preferred position-dependent consensus indexes were calculated from siRNA sequence, as shown in Supplementary Materials [see Additional file 3].

The distributions of preferred and avoided position-dependent consensuses within the efficient and inefficient sets of siRNAs were substantially different [see Additional file 1]. Nucleotide preferences are most pronounced at positions 1–3, 13–14 and 17–19 (Figure 1). In positions 1–3 (three terminal positions for the antisense strand) nucleotides U and A are preferable, especially U in the first position. Nucleotide G is depleted in all three terminal positions at the 5' end of the antisense strand, and C is also depleted in the first position. Frequencies of U are enhanced and frequencies of G are reduced in positions 13–14. Positions 17–19 are depleted in nucleotide A, at the same time nucleotides C and U are preferred in positions 17–18, and for position 19 nucleotides C and G are preferable.

miRNAs have the same nucleotide biases at the 5' end of the mature sequence. The frequency of nucleotide U is increased in first positions of mature strands of miRNAs, just as in the antisense strand of efficient siRNAs. There are strong requirements for elevated AU content in the first position and GC-richness at the 3' end of the antisense strand of siRNA duplexes.

### Thermodynamic profile and free energy differences (ΔG) between 5' and 3' ends of siRNA and miRNA duplexes

**Figure 2:**
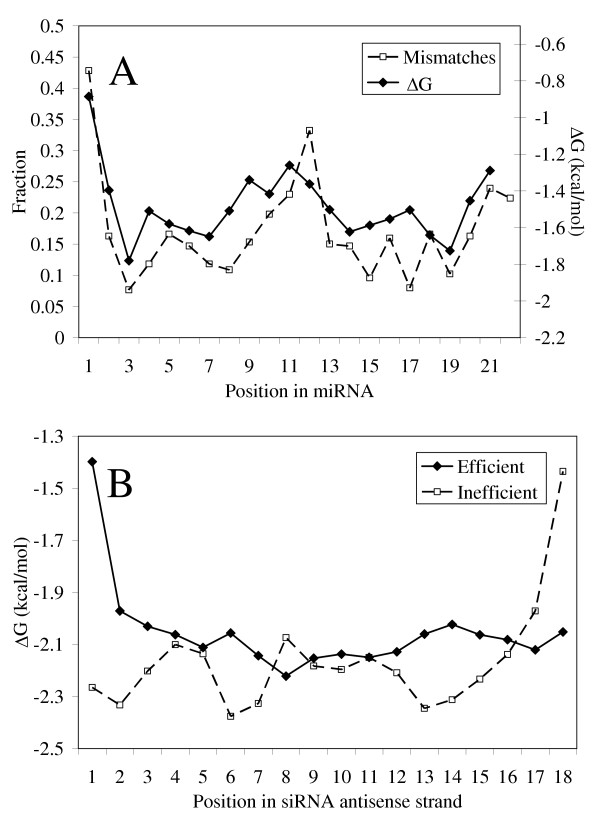
**Thermodynamic stability profiles for miRNAs and siRNAs**. A) miRNA stability profiles and distributions of mismatches in miRNA precursor structures for 22 nucleotide lengths. B) Stability profiles of efficient and inefficient siRNAs.

We calculated thermodynamic profiles for sense-antisense duplexes of efficient and inefficient siRNAs and for published miRNA sequences. The distribution of the matches for potential duplexes in the precursor molecules for mature miRNAs of 22 nucleotide lengths and the profile of free energy of pre-miRNA duplexes are presented in Figure 2A. Similar results were obtained for miRNA precursors of 21 and 23 nucleotide lengths (data not shown). The observed high values of free energy in two regions (nucleotides 1, 9 – 13) can be explained by mismatches in the formation of pre-miRNA secondary structures. Our data suggest that nucleotides 3–4, 6–8, 14–15 and 19 more frequently interact complementarily in miRNA precursors. In contrast, position 1 and positions from 9 to 13 frequently are unpaired (Figure 2A).

Thermodynamic stability profiles for efficient and inefficient siRNAs are presented in Figure 2B. Duplex stability in positions 1–2, 6, 13–14 and 18 are different for efficient and inefficient molecules (see Table 1). A significant correlation between free energy of base pairs in these positions and the efficiency of silencing (Table 1) suggests that these positions can be important for siRNA design. Efficient siRNA duplexes are characterized by less stable interaction at the 5' ends and more stable interaction at the 3' ends of antisense strands, the opposite of inefficient siRNA duplexes. Overall, thermodynamic stability in efficient siRNA duplexes at the 5' end and the 3' end follow the same pattern as in a mature miRNA duplex.

Our data show that nucleotides 17–19 in mature miRNAs ought to be more thermodynamically stable than the others and also frequently interact complementarily in miRNA precursors. Regions 18–19 in efficient siRNAs have a higher C/G-content and are thermodynamically stable as well. Efficient siRNAs have the same low stability in the first pair of the antisense strand as mature miRNAs (Figure 2A–B). Also, the free energy difference between 5' and 3' ends is similar for efficient siRNA antisense strands and mature miRNAs.

The distributions of calculated free energy differences between 5' and 3' terminal nucleotides in siRNA and miRNA duplexes are presented in Supplementary Materials [see Additional file 1]. This difference is an important parameter for efficient siRNA prediction. These data are in agreement with experimental evidence that the difference in free energy (ΔG) can dramatically affect the efficiency of integration of the antisense and sense strands into RISC [3]. To optimize this parameter, we calculated the ΔG difference of duplex formation at the 5' and 3' ends of siRNA for 2, 3, 4, and 5 terminal nucleotides and compared correlation coefficients between these values and siRNA efficiencies (Table 3). We calculated these values for two different experimental data sets measuring integration of the antisense and sense siRNA strands into RISC [3,11]. In all cases, the difference in siRNA end stability for terminal dinucleotide pairs had the highest correlation coefficient with siRNA efficiency (Table 3). This parameter also showed the best discriminating power for prediction of the ratio of antisense versus sense strand incorporation into RISC. In our data set, we observed the same pattern.

**Table 3:** Correlation of thermodynamic stability difference of the 5'/3' ends of siRNA duplexes with siRNA activity (AS) and with the ratio between the antisense strand and sense strand activities (AS/SS). Differences in the stability of the 5' and 3' ends were calculated for 2 (Diff2), 3 (Diff3), 4 (Diff4) and 5 (Diff5) terminal nucleotides in siRNA duplexes.

Data set	Activity or ratio	Correlation coefficients				Reference
			
		Diff2	Diff3	Diff4	Diff5	
28 siRNAs	AS	-0.245	-0.196	-0.171	-0.103	Hu et al. [11]
28 siRNAs	AS/SS	-0.384	-0.356	-0.368	-0.278	Hu et al. [11]
18 siRNAs	AS	-0.870	-0.869	-0.748	-0.767	Schwartz et al. [3]
18 siRNAs	AS/SS	-0.629	-0.544	-0.471	-0.453	Schwartz et al. [3]
653 siRNAs	AS	-0.356	-0.332	-0.292	-0.228	This work

### Thermodynamic characteristics of competitive duplex structures

As shown previously, duplex stability is very important for hybridization and antisense oligo-RNA interaction [27,28]. It is hypothesized that oligo intra- or intermolecular structures can compete with oligo-target duplex formation which may result in low hybridization efficiency. Extensive secondary structure of the target can also limit this efficiency. We addressed the thermodynamics of the relative stability of siRNA antisense strand-target duplexes, siRNA antisense strand-intra duplexes and inter-molecular self-structures of the antisense strand of siRNA, and found that thermodynamic considerations improve the selection of efficient siRNA targets in mRNAs. The following thermodynamic features are significantly different for efficient and inefficient siRNAs and are important for optimal target prediction: (i) Gibbs free energy of sense-antisense duplexes, (ii) free energy of siRNA self-structure and free energy of siRNA bimolecular interaction, (iii) mRNA secondary structure and free energy of siRNA antisense strand affinity to mRNA targets, (iv) frequency of potential siRNA targets in mRNA. The correlation coefficients and the results for *t*-tests are presented in Table 1.

The average of duplex free energy for efficient (<10% residual transcript level) and inefficient (>70%) siRNAs are different (*t *= 4.9, *p *< 8.6e^-7^), and it is significantly lower for efficient siRNAs. The distributions of Gibbs free energy for the two subsets of siRNAs are different, and the distribution for pre-miRNA duplexes is closer to the distribution for efficient, than inefficient siRNAs [see Additional file 1].

There is a significant correlation between the silencing efficiency and stability of secondary structures of the antisense strand of functional siRNAs (Table 1). For most of the efficient siRNAs, the ΔG of self-interaction is close to 0. Stability of self-interaction of the mature miRNAs is also high and ΔG values of self-interaction in mature miRNAs are significantly higher than those for randomly shuffled oligos with the same nucleotide content (data not shown). Likewise, there is a correlation between stabilities of both oligo-intra and inter-molecular self-structures [see Additional file 6].

RNAs form stable secondary structures through Watson-Crick and wobble G-U base pairing. The single-stranded regions are likely to be more accessible for RNA-targeting nucleic acids through base pairing interactions than double-stranded structures. We found significant negative correlation between ΔG of the local secondary structure of mRNA target site and the silencing activity of siRNAs (Table 1).

Another parameter important of siRNA efficiency is the frequency of potential targets for siRNA in the mRNA, i.e. mRNA sites capable to form stable duplexes with oligoprobes. We estimated the frequency of potential siRNA targets in the mRNA sequence using thermodynamic parameters [29] and stability threshold of duplex formation of -18 kcal/mol, which was determined from the analysis of RNAi data. Results of this analysis are presented in Table 1.

### Computational models

We calculated stability *S*_4 _for 18 parameters as described in Materials and Methods, with the results presented in Table 1. We chose the tight cluster of parameters with *S*_4 _≈ 0.065 as the cut-off for stable parameters. At the same threshold, there is a noticeable drop in *R *values. The stability values for the best 12 parameters are presented in bold in Table 1.

**Figure 3:**
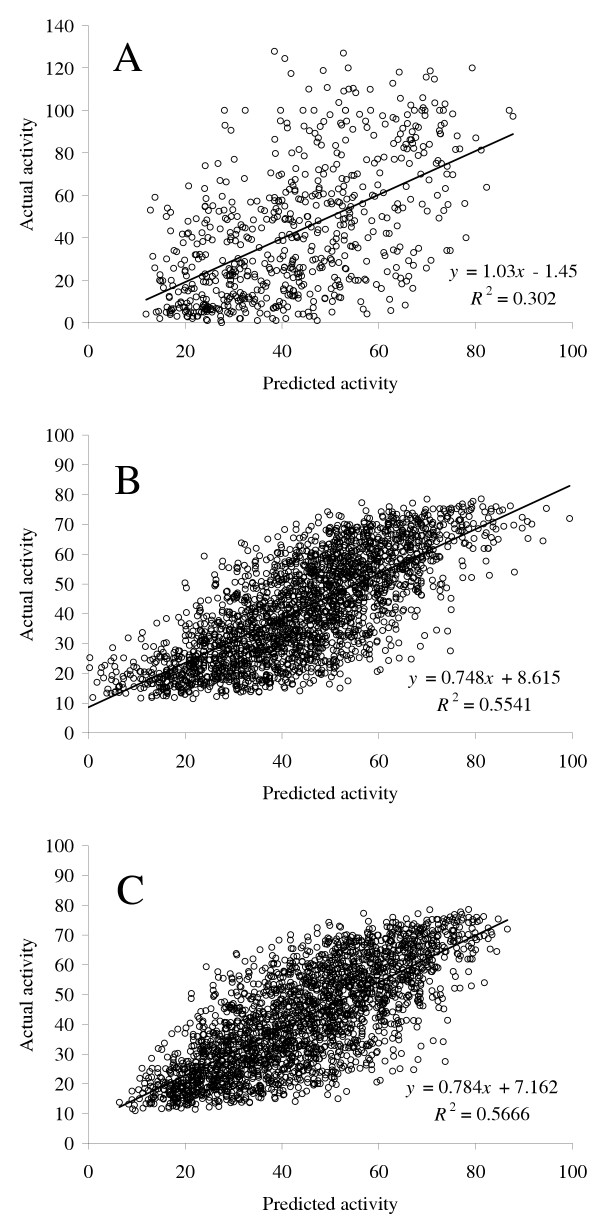
Plot of neural network predictions versus the actual siRNA activity for 653 siRNAs with a broad concentration range (A). Plots of linear regression (B) and neural network predictions versus the actual siRNA activity for the siRNA validation set (C).

Out of these 12 stable parameters, we identified the set of four stable parameters (Table 1, shown in italic) possessing the best predictive power on the training set (*R*^2 ^= 0.301). We used an optimized set of four stable parameters (three parameters characterizing siRNA features and one parameter characterizing the number of potential target sites on mRNA). Cross-validation predictions of a 4 × 2 × 2 × 1 network with ALPHA = 2.0 and 150 epochs of training yielded *R*^2 ^= 0.301. The corresponding scatter-plot of predicted versus actual values for 653 siRNAs with a broad range of concentrations is shown in Figure 3A. To estimate relative contributions of these parameters to the classifier performance, we measured neural network performance without each of these parameters: summarized position-dependent consensus (*R*^2 ^= 0.172), avoided dinucleotide content index (*R*^2 ^= 0.288), ΔG difference between 5' ends of sense and antisense strands (*R*^2 ^= 0.271), and the number of potential target sites in the target mRNA (*R*^2 ^= 0.284). These figures indicate that summarized position-dependent consensus is the most important parameter, which dramatically improves siRNA target prediction.

One of the parameters – the frequency of potential targets in the mRNA – could not be meaningfully computed on the validation data set. Most of the 653 training siRNAs had their efficiencies evaluated against complete mRNA sequences. In contrast, the Novartis data set used a short piece of cDNA inserted into a plasmid as the target. We chose to avoid complicated approximations for this fourth parameter, and used 3-parameter models on the validation set.

We used a multiple linear regression as our baseline modeling technique. We predicted validation set (2431 siRNAs) efficiencies using the 3-parameter model produced on the training set. The plot of predicted versus actual efficiencies for validation siRNAs is shown in Figure 3B. We had no reason to expect that a linear model would work best on our data. Thus, we also applied neural networks, which would be able to fit any reasonable function.

**Figure 4:**
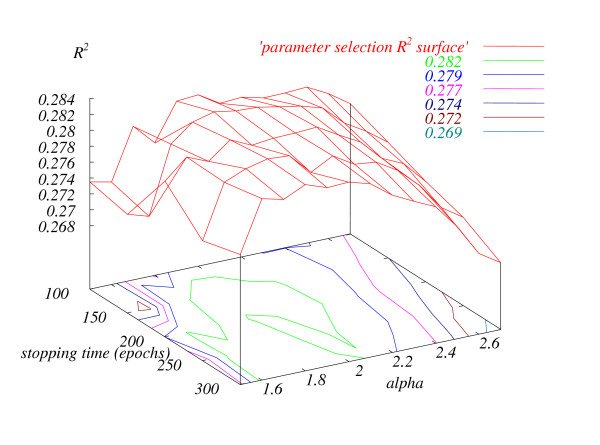
**The dependence of *R*^2 ^on the two neural network training parameters, ALPHA and stopping time**. The lower part of the figure shows the contours of the surface. For instance, the inner-most light green shape delineates the parameter values giving *R*^2 ^≥ 0.282.

During training, we experimented with a number of 18-parameter neural network configurations, as described in Materials and Methods. Table 4 presents the resulting *R*^2 ^values. *R*^2 ^varied very little with the network layout (± 0.02) for all 1- and 2-hidden layer networks. We then optimized the parameters for the marginal winner 18 × 3 × 2 × 1, as described in Materials and Methods. Figure 4 presents the optimization landscape. Again, there was very little variability for reasonable values of ALPHA: ± 0.02 for 1.6 < ALPHA < 2.1. With the 18 × 3 × 2 × 1 network, 150 epochs, and ALPHA = 2.0, the training set 18-parameter cross-validation *R*^2 ^was 0.283. The details of the trained neural networks are presented on the NCBI ftp site at .

**Table 4:** The value of R^2 ^(50-split average) for neural networks after a given parameter is removed. The standard deviation of the 50-split average is also shown.

Neural Networks configuration	*R*	*R*^2^	std. dev.
18 × 1	0.5222	0.2727	0.0073
18 × 2 × 1	0.5310	0.2820	0.0066
18 × 3 × 1	0.5310	0.2820	0.0062
18 × 4 × 1	0.5306	0.2815	0.0065
18 × 6 × 1	0.5302	0.2811	0.0061
18 × 8 × 1	0.5296	0.2805	0.0064
18 × 2 × 2 × 1	0.5322	0.2832	0.0058
18 × 3 × 2 × 1	0.5324	0.2834	0.0061
18 × 3 × 3 × 1	0.5322	0.2832	0.0059
18 × 4 × 3 × 1	0.5321	0.2831	0.0063
18 × 6 × 2 × 1	0.5315	0.2825	0.0058
18 × 2 × 2 × 2 × 1	0.4041	0.1633	0.0704

We found that it is preferable (because of speed, simplicity, and better performance) to use the 3- or 4-parameter (when mRNA features could be estimated) sets in practice. For the validation experiments with 3 and 4 parameters, we simply borrowed the 18-parameter settings. With 18 parameters, the network configuration and parameters had little effect on performance, and so further optimizations would likely not be worthwhile.

We trained a 3 × 2 × 2 × 1 network with ALPHA = 2.0 for 150 epochs on the 653 siRNA set, and predicted efficiencies for the siRNA validation set. The model's scatter-plot of predicted versus actual values is shown in Figure 3C.

**Figure 5:**
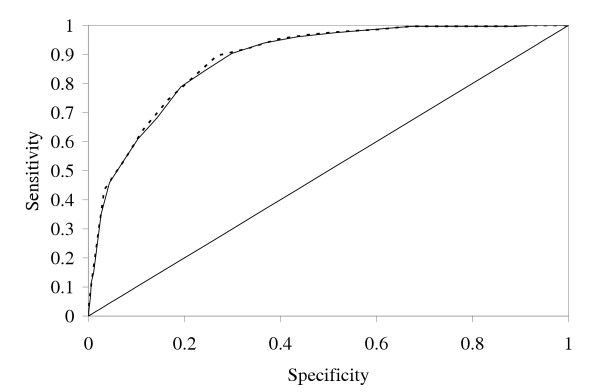
**Combined ROC curves with 20% residual activity thresholds**. The dotted line shows the curve for the neural network classifier, while the solid line shows the curve for the linear regression classifier.

For practical purpose one often needs a classification decision – will this siRNA be active or inactive? We investigated two thresholds of silencing activity: 20% and 30% residual mRNA expression. We computed the ROC (receiver operating characteristic) curves from the 2431 siRNA validation set output of the linear regression and the neural network model, as described in Materials and Methods (Figure 5). The areas under the curves are 0.882 (20%) and 0.865 (30%) for linear regression, and 0.886 (20%) and 0.867 (30%) for the neural network. The classifiers from the two models are practically indistinguishable, but in both cases the 20% predictions are a little better.

In practice, it is not essential to find every active siRNA, just some active siRNA with as few failures as possible. For high-specificity predictions, a suitable point on the 20%-threshold ROC graph might be that with 88.5% specificity and 64.2% sensitivity. For 30%, one might choose 87.4% specificity and 65.3% sensitivity.

**Figure 6:**
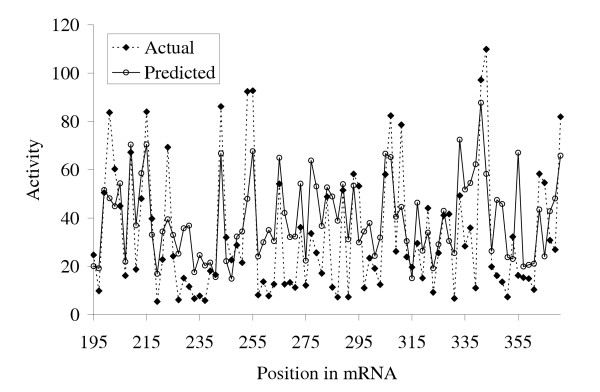
The profile of predicted (this study) and experimentally determined activities [1] for sliding window of 19 bases along human cyclophilin B (hCyPB) mRNA target sequence (GenBank accession number: M60857).

It is expected that this set of 3 parameters can substantially improve the efficiency of prediction on any dataset. Importantly, all of these parameters can be computed efficiently and can be easily used for large scale siRNA target prediction at the transcriptome level. Thus, it makes possible to offer an *in silico *prediction of efficient targets for all known mammalian transcripts. We calculated these stable siRNA parameters for ~22600 human transcripts, and these data are available in Supplementary Materials. The profile of predicted and actual activities for sliding window of 19 bases along a sequence of human cyclophilin B mRNA (*R*^2 ^= 0.448) is presented in Figure 6. It is seen, that our prediction corresponds well to experimentally determined siRNA activities.

We tested the sets of 18 parameters and the four best parameters on an additional set of 32 siRNAs which we did not include in our siRNA database and did not analyze before. The siRNAs in this set were tested in experiments at low concentrations [see Additional file 7]. This analysis showed that both the 18-parameter model and the 4-parameter model produced good predictions (*R*^2 ^= 0.258 and *R*^2 ^= 0.281, respectively) for experimental set of data obtained at low siRNA concentrations of siRNAs.

### A note about cross-validation

When working with out 653 siRNA training data set, we used a non-overlapping cross-validation technique. The goal was to make sure that there were no highly similar siRNAs between the test and training data sets. We ran both the linear regression and neural network models using standard 7-fold cross-validation as well. The resulting *R*^2 ^values with our 3 parameters were approximately 0.01 larger: 0.288 rather than 0.276 for linear regression, and 0.298 instead of 0.285 for neural networks. Thus, having partial overlaps between test and training siRNAs may lead one to overestimate generalization performance.

## Discussion

We analyzed a set of siRNAs (653 sequences) with known efficiencies from different sources using some novel prediction parameters and published parameters from literature [26]. This data set included experiments performed with a broad range of siRNA concentrations, substantially biased towards the high end (over 300 siRNAs tested at 100 nM concentrations). In contrast, the 2431 validation set was evaluated entirely at 50 nM concentrations. We analyzed 18 thermodynamic and composition parameters, which can, to some extent, separate efficient and inefficient sets of siRNAs.

Dinucleotide content is a better predictor of efficiency than mononucleotide content; this suggests that both nucleotide content and base stacking are important for efficient silencing. Content frequencies, especially for preferable dinucleotides, differ in siRNAs and miRNAs [see Additional files 4 and 5]. Likely, these differences reflect functional differences between suppression and cleavage mechanisms in RNAi. Previous attempts to derive position-specific siRNA consensus were based on the analysis of nucleotide frequencies in small sets of efficient siRNA and yielded controversial results [13,30]. Our consensus is based on correlation and t-test analyses of large siRNA dataset, accounting for both significantly preferred and avoided nucleotides at all sequence positions. This approach allows quantitative estimation of position-specific nucleotide preferences and avoidances in a large data set of efficient and inefficient siRNAs. Our data coincided with previously reported data on base preferences (position 1 for A and U, position 10 for A, and position 19 for C) and dinucleotide content [31,32]. Efficient siRNAs and miRNAs share similar sequence features at the 5' end of the antisense strand. Comparison of the 3' terminal positions for siRNA and miRNA sequences is made difficult by the differences in miRNA lengths and the lack of a good miRNA consensus there. Nevertheless, similar trends such as elevated G or G/C content are apparent in this region. An opposite tendency for U/A and C/G nucleotides at the 5' and 3' ends of the antisense strand in efficient siRNAs is in agreement with published results on asymmetry of siRNA duplexes [1,3], which is related to siRNA efficiency [15].

Our data indicate that the antisense strands of efficient siRNAs are U-rich, and therefore are likely to interact with polypurine tracks in mRNA targets. It was demonstrated that polypurine RNA tracks complexed with pyrimidine-rich oligonucleotides are recognized by RNase H [33], and the catalytic domain of RNase H resembles a key functional region in the Argonaute protein [25,34]. These observations suggest that siRNA-mediated mRNA cleavage has some similarity to mechanism of RNA degradation by RNase H. In contrast, miRNAs are G, U-rich. Differences in nucleotide composition between siRNAs and miRNAs may reflect different requirements for mRNA cleavage and mRNA translational repression.

Thermodynamic profiles for efficient and inefficient siRNAs (averaged for pentanucleotides) have been discussed earlier [12]. It was shown that siRNA duplexes are functionally asymmetric and only one strand is preferentially incorporated into the RISC [3]. The relative and absolute binding energies of the 5' antisense and 5' sense strands determine which strand enters the RISC complex. Preferential uptake of one strand into RISC based on the thermodynamic stability of siRNA duplex is an important criterion for design of efficient siRNAs. The siRNA's thermodynamic properties must be such that the RISC prefers the incorporation of the strand that is complementary to the intended target site. Different authors calculated siRNA internal stability profiles using scanning windows of different length: pentanucleotide [1] tetranucleotide [3] and trinucleotide [16]. In our study we optimized the length of the scanning window for assessment of the functional asymmetry between the sense and antisense strands. We found that free energy differences calculated for two terminal dinucleotides of both ends in siRNA duplexes according to the nearest neighbor method [29] possessed the best discriminatory power for distinguishing between the antisense and sense strands of siRNAs and their ability to integrate into RISC. We conclude that thermodynamic profiles and free energy differences between the 5' ends of sense and antisense strands are important for RNAi efficiency and, most likely, are crucial for the optimal siRNA design. Our data are in good agreement with the results for miRNA analysis [16], where it was shown that the two terminal nucleotides best distinguish between the miRNAs and its complementary strand.

To understand the thermodynamic requirements for duplex unwinding and strand incorporation into RISC, we compared the thermodynamic profiles and match-mismatch patterns of interactions in pre-miRNA molecules (Figure 2A). Patterns of duplex stability follow the patterns of nucleotide mismatches in pre-miRNAs of different length, the 5' end of the antisense strand and the central bases of the duplex being generally less stable than the 3'end of the same strand (Figure 2A). Stability profiles for efficient siRNAs at the ends of duplexes show the same tendency as for pre-miRNAs (Figure 2B). We suggest that the positions' tolerance to mismatches in pre-miRNA secondary structure is important for duplex unwinding and incorporation into RISC. This pattern of mismatches observed in pre-miRNAs may be used in the construction of a synthetic hairpin (fork) siRNAs to facilitate duplex unwinding and strand assembly into RISC.

Target recognition is highly sequence specific process. However, not all positions of a siRNA contribute equally to target recognition. From the analysis of miRNA and efficient siRNA thermodynamic profiles and from their content comparison we can infer that the central region of siRNA antisense strands is probably important for target recognition or cleavage. This suggestion is in agreement with experimental data that mismatches in the central part of the siRNA duplex (nucleotides 5 – 12) prevent target RNA cleavage [35,36]. In the case of miRNAs, nucleotides 2 to 7 seem to be the most important for translational suppression [37-40]. Recent experimental studies show that there are two classes of miRNA target sites: 5' dominant sites with sufficient complementarity to the miRNA 5' end, and 3' compensatory sites with strong 3' paring and insufficient 5' paring [41].

We believe that the mosaic pattern of stability in siRNA duplexes is a compromise between the need for weak interaction in positions important for duplex unwinding and RISC incorporation, and the requirement for strong interaction with mRNA targets. Thus, different thermodynamic properties of specific siRNA regions are crucial for efficient silencing. Therefore, detailed analysis of thermodynamic profiles is more helpful for siRNA design than estimation of total free energy of duplexes formation.

Another important issue is the number of potential target sites that possess substantial complementarity to a siRNA. The presence of multiple sites in the target mRNA can cause a more efficient degradation due to a cooperative effect or an increased probability of the accessible single strand conformation of the target. To address this problem, we introduced a parameter which determines frequencies of potential (off-site) targets with substantial complementarity and low free energy of duplex formation between a siRNA and the target mRNA. On the other hand, substantial complementarity between a siRNA and untargeted mRNAs can result in off-target repression, which is a major issue in the field of RNAi.

Our results demonstrate that competing base pairing interactions (siRNA self structures, siRNA duplexes, and secondary mRNA structures) affect the efficiency of siRNAi, and that the features of the siRNAs (or miRNAs) themselves are important for the efficient RNA interference. The secondary structure of the target RNA has been shown to be important for siRNA-mRNA interaction [18-20]. Target accessibility has long been established as an important factor for the potency of antisense oligonucleotides and trans-cleaving ribozymes. Selection of mRNA target sites and siRNA sequences that are free from stable secondary self-structures helps produce better results in siRNA design [42]. However, calculation of the target secondary structure and accessibility is computationally expensive (quadratic time or slower) and is not practical for large-scale predictions. For the needs of transcriptome-scale RNAi projects, we selected the short list of stable parameters that contain no mRNA features and produce reliable results on every subsets of our database. These selected parameters were used for siRNA target prediction in human transcripts. The difference from previous NN applications and the novelty of our work is that our approach is based on both thermodynamic and composition features. Calculations of full position-dependent consensus and dinucleotide content indexes together with thermodynamic differences between 5' ends of the sense and antisense strands of siRNA duplexes improve siRNA design.

We investigated the ability of neural network models to improve siRNA target prediction over traditional linear models. The neural network regression results do not differ substantially from those of the linear regression. Our model has few parameters, and none of them are strongly non-linear, which would account for this similarity. Correspondingly, the performance of our threshold-based classifier is almost identical for neural networks and linear regression. Our classification results have room for improvement. Firstly, for a classifier based on regression results, the regression scoring function should be biased to encourage higher accuracy on active siRNAs. Secondly, at the expense of a more complex training process, a dedicated neural network classifier could be used.

## Conclusion

In this paper we showed that the properties of the siRNAs themselves are important for RNA interference. Determination of the preferred and avoided position-dependent consensus of the antisense strand of siRNA duplexes is an important step in the efficient siRNA design. The 5' ends of antisense strands of efficient siRNAs are U-rich and possess a content similarity to the pyrimidine-rich DNA oligonucleotides interacting with the polypurine RNA tracks that are recognized by RNase H. Also, the introduction of mismatching bases at certain positions of siRNA duplex may be beneficial for duplex unwinding and incorporation of the antisense strand into RISC. Some of our mRNA features, though expensive to compute, can slightly improve our model. Analysis of thermodynamic and compositional features of siRNAs based on computational models is a promising approach for transcriptome-scale prediction of the target sites for efficient RNA interference. Transcriptome-wide analysis of optimal mRNA target prediction has a clear potential for uncovering previously undetected features in efficient siRNAs.

## Methods

### Data and sources

The siRNA set was compiled from published experiments targeting 52 distinct mRNAs. The effectiveness of each siRNA sequence was judged by its gene silencing activity as shown in the Results section of the publication [see Additional file 4]. Most of reported activity values are averages of duplicate measurements at a constant oligonucleotide dose expressed as percent of untreated control. Reported activity values range from 0 (complete knock-out) to = 100% (no effect). Based on reported activity, siRNAs were then divided into three groups: (i) siRNAs that induced more than 70% gene silencing (a total of 295), (ii) siRNAs that induced less than 30% gene silencing (130 in total), and (iii) the remaining sequences in the collection. Our database of siRNAs is presented in Supplementary Materials [see Additional file 4]. The database consists of two subsets of siRNAs produced by Dharmacon and Amgen Incorporated (298 siRNA duplexes) and other sources (355 siRNAs). The total number of analyzed siRNAs with experimentally determined silencing efficiency is 653. Every entry in the database consists of an oligonucleotide sequence, the target mRNA GenBank sequence accession number, the sequence and coordinates of the intended mRNA target region, miRNA silencing efficiency for that mRNA, and the publication source. The resulting models were tested on the 2431 siRNA dataset recently published by Huesken et al. [24].

The siRNAs in the database were screened for the following criteria. (i) Gene expression was measured after siRNA application relative to untreated control. (ii) At least 5 siRNAs were assayed for a given target mRNA. (iii) The nucleotide sequence of the antisense strand has a perfect complementary target site in the mRNA sequence. Analysis was performed for 19-nucleotide oligonucleotides and did not include the overhangs at the 3'-ends. The important technical details of the corresponding gene silencing experiments are shown in the Material and Methods sections of each publication. We analyzed sets of miRNAs available at  for *Homo sapiens*, *Mus musculus *and *Rattus norvegicus*.

We calculated a number of thermodynamic features such as: ΔG values per nucleotide pair that are relevant to stabilities of sense-antisense siRNA duplexes, siRNA antisense strand intra-molecular structure stability, siRNA antisense strand inter-molecular dimer stability, local target mRNA stabilities, and stabilities of each two neighboring base pairs in the siRNAs sense-antisense duplexes. These characteristics were calculated using a nearest neighbor model (see Software). The following section describes the modeling methods we used.

### Training and validation sets

We used only the 350 siRNAs from 653 siRNA training set when creating composite parameters such as the summarized position-dependent consensus. Parameter selection and model optimization were also restricted to the 350 siRNAs initially and then applied to the complete training set (653 siRNAs).

On the 2431 siRNA validation set, we made just two efficiency predictions (using models obtained from the training set): one with linear regression, and one with the best neural network model. We also used these validation set predictions to generate ROC curves for the corresponding classification models.

### Parameter selection

We used a number of parameters described in the literature [23], and supplemented them with a number of new ones. In all, we had a list of about 150 parameters, which is too large for effective analysis. We used two criteria to reduce this number: significant correlation with activity and stability of the correlation. Both criteria were evaluated on the training set only. Overall block scheme illustrating siRNA feature selection approach is presented in Supplementary Materials [see Additional file 2].

We required that parameters have a correlation of at least 0.1 with siRNA efficiency, and that this correlation be significant at the 0.05 level. This left 18 parameters, as detailed in Results.

Since our training set is very heterogeneous, combining many experiments and slightly different protocols, we had the opportunity to select those parameters which are most universal. To do this, we split our data set into *n *parts (*n = *4 and 10), and for every part and parameter computed the correlation coefficient. Taking 1000 such splits, we computed *S*_*n*_, the standard deviation of *R *for every parameter. We used this stability *S*_*n *_value as an indicator of how much the parameter's predictive power depends on the choice of the particular subset of the data.

### Linear regression model

In our analysis, we used regression, rather than classification models, since they provide more information, are more flexible, and are easier to evaluate. We performed multiple linear regression analysis on our sets of 18 parameters, with cross-validation as described below. We used scripts written in the Octave (MATLAB work-alike) language. The scripts are available by request.

### Neural network model

In all neural network experiments, we used the Stuttgart Neural Network Simulator (SNNS) V4.2, available at the SNNS website . We chose fully-connected multi-layer feed-forward networks (created using the BIGNET->GENERAL tool in SNNS). Such a network starts with a layer of input neurons (one per feature), which is connected to a hidden layer, with a connection going from every input to every hidden neuron. The first hidden layer may be fully connected to another hidden layer, and so on. The last hidden layer is fully connected to the output neuron. Each neuron integrates information from the incoming connections, and outputs it to the next layer. The integration is a two-step process. First, the inputs are summed with neuron-specific coefficients (which are chosen adaptively during learning). Then, the sum is transformed with an activation function (which has its own adaptable coefficient), and the result is output. We used logistic activation functions (default SNNS setting) on all neurons.

We achieved the best results with resilient propagation (RProp). The algorithm uses the following parameters: (i) the initial learning step size, to which generalization performance is insensitive, and which we set to 0.1, (ii) the maximum learning step size, also unimportant for generalization, and set to 10, and (iii) the weight decay parameter ALPHA, which was determined by a simple search (See the subsection "Training parameter selection"). We were unable to get better performance with other algorithms or a linear, rather than logistic, output function (results not shown).

The training process consists of epochs. Each epoch SNNS trains the network on all points of the training data set (as given by the cross-validation procedure) in random order (set the SHUFFLE option in SNNS). Every epoch, we also modified each coefficient in the network by ± 0.1% (SNNS option JOG weights with setting 0.001), since we found that this helps reduce the variation in performance by pushing the network out of local minima.

An important decision during training is when to stop training the network. An under trained network is a poor fit to both the training and the validation data set. Excessive training is time-consuming, and the network might over fit the training data set and get worse on the validation set. Although a correct setting of the ALPHA parameter in RProp reduces over fitting, it is still important to choose a good stopping time. A traditional approach called "early stopping" is to split the training set into a training subset, and a small "test" subset, and stop when the error on the test subset reaches a minimum. We did not do this for two reasons. Firstly, it reduces the training set, and increases the variation in the results (from the choice of the split into training/test sets). Secondly, when ALPHA is set properly, the validation error tends to be nearly monotonically decreasing, making early stopping unnecessary. Instead, we searched for an optimum fixed number of epochs, after which to stop (see "Training parameter selection").

We tested the cross-validation performance of the following network geometries (input neurons = 18 × hidden layer × hidden layer × ... × output neurons = 1): 18 × 1 (logistic regression), 18 × 2 × 1, 18 × 3 × 1, 18 × 4 × 1, 18 × 6 × 1, 18 × 8 × 1, 18 × 2 × 2 × 1, 18 × 3 × 2 × 1, 18 × 3 × 3 × 1, 18 × 4 × 3 × 1, 18 × 6 × 2 × 1, 18 × 2 × 2 × 2 × 1. Comparing the number of training coefficients to the number of training points, some overfitting might be possible for an 18 × 8 × 1 network. However, given the behavior of RProp, it is not likely: the algorithm slows down the learning process gradually in a way that helps to avoid overfitting. For network selection, we used the reasonable parameters ALPHA = 1.8 and 250 epochs of training, which were determined by hand experimentation in SNNS on a 200-siRNA sample of the data. We found that all 18-parameter 1- and 2-hidden layer networks performed very similarly, and so only tested the 4 × 2 × 2 × 1 network in the 4-parameter case. Scripts for many of the above tasks are available by request.

### Data scaling

In order to keep features with large numerical scales (e.g. varying from -30 to -5) from overshadowing features with small numerical scales (e.g. 0 to 0.2), we linearly rescaled all features to the range [0, 1]. Because our output neuron uses a logistic activation function, it is biased against producing outputs close to 0 or 1. To help with this problem, the efficiency values were linearly rescaled to [0.25, 0.75].

### Classification and ROC computation

Neural networks originated as a classification technique. A classification decision in such a network is made by putting a threshold function in the output neuron. The network would then be trained to optimize some measure of performance (% error, or perhaps another measure, favoring higher sensitivity or higher specificity). However, for different purposes, one may wish to define an active siRNA as one with 5%, 10%, 20%, or even 30% residual activity. For every such choice, one would have to re-train the network.

A threshold on regression output can produce a classification decision too. A regression model enables us to avoid retraining – we only need to choose a threshold for the predicted value, a much quicker computation than network training. We computed ROC (receiver operating characteristic) curves for classifiers of this type, as follows.

First, we separated the validation set predictions into "active" and "inactive" based on the actual silencing efficiency. For every predicted activity threshold in this list: 3%, 6%, 9%, ..., 96%, 99%, we repeated the following steps. We marked each prediction "predicted active" or "predicted inactive", based on the current threshold. The total number of "active, predicted active" gave us the true positive (TP) count. Similarly, we obtained FP, TN, FN counts, and from them calculated Sensitivity = TP/(TP+FN) and Specificity = TN/(TN+FP). The result is 33 pairs of the form (1 – Specificity, Sensitivity), which give the ROC curve.

### Cross-validation and performance

When choosing or optimizing models on the training set, we used *n*-fold cross-validation, a standard method for evaluating model generalization. Cross-validation randomly splits the data set into *n *equally-sized subsets. Then, one trains, in turn, on each subset set of *n*-1, validating on the remaining one. The validation predictions from the *n *models combine to make a prediction for every data point. Using these predictions, we compute the coefficient of determination *R*^2 ^= (*Actual Variation – Error*)/(*Actual Variation*), where the actual variation is ∑_*i*_(*Actual efficiency*_*i *_- *Average efficiency*)^2^, and the error is ∑_*i*_(*Actual efficiency*_*i *_- *Predicted efficiency*_*i*_)^2^. Thus, *R*^2 ^reflects the percentage of variation in efficiency explained by our model. If the predictions came from a non-cross-validated linear regression, this *R*^2 ^would exactly match the square of Pearson's correlation coefficient.

Standard cross-validation can overestimate the generalization performance because substantial numbers of the siRNAs in our data set overlap. If a training siRNA overlaps with a validation one, a good prediction for the validation siRNA does not demonstrate generalization.

To address this issue, we designed the following cross-validation scheme. Instead of subdividing into parts uniformly at random we developed a tool which splits the database using the following algorithm: (i) allocate *n *empty buckets. While there are unallocated siRNAs, do the following steps: (ii) find the bucket *i *with the smallest number of elements. (iii) Pick an unallocated siRNA *s *uniformly at random, put it into *i*. (iv) All siRNAs overlapping *s *are placed into *i*, as are siRNAs overlapping those siRNAs, etc. The result of the algorithm is *n *parts of nearly equal size, with no pair of overlapping siRNAs that is split between two parts. This algorithm is preferable to the standard minus-mRNA approach in our case because some mRNAs have many more siRNAs than others (100 siRNAs versus 5). The subsets only stay roughly equal up to *n *= 7, because there are some overlapping chains of ≈653/7 = 93 siRNAs long.

For *n *> 7, we tried a similar version of the algorithm, which discarded a fraction of the data in order to break up long overlapping chains. Using this method with *n *= 20 or 30 helped variability in *R*^2 ^rather little, but slowed computations a lot (results not shown). Hence, we did all cross-validation using the simpler algorithm above with *n *= 7. To reduce variation, cross-validation results were typically averaged over 50 such random splits. We also compared the results of our non-overlapping cross-validation algorithm with standard random cross-validation.

### Training parameter selection

Our neural network training procedure had two parameters with the potential to affect generalization performance. We selected the best pair using the following procedure. We picked a set of 50 random cross-validation splits; this set remained fixed for the entire procedure. We then varied ALPHA from 1.5 to 2.7 in steps of 0.1, and tried stopping times from 100 to 300 in steps of 50 epochs. For every pair of parameters, we output the 50-split average *R*^2 ^and its standard deviation.

### Software

The tools and scripts used in producing and evaluating these models are available by request. We use a combination of the C programming language, the **Bash **shell scripting language , GNU **Octave **scripting , and SNNS v 4.2 Batchman (see Neural Networks). The tool set was developed on a **Gentoo GNU/Linux **system .

Calculations of a list of thermodynamic values such as: ΔG values that are relevant to stabilities of sense-antisense siRNA duplexes, siRNA antisense strand intra-molecular structure stability, siRNA antisense strand inter-molecular dimer stability, local target mRNA stabilities, and stabilities of each two neighboring base pairs in the siRNA sense-antisense duplexes were done with the OligoTherm program. OligoHybrid is a tool for calculation of potential targets of complementary interactions between two RNA molecules. Calculations of potential secondary structures for RNA molecule and estimation of the free energy of the local secondary structure and prediction of oligonucleotide affinity to nucleic acid targets were performed with the RNApack program. These programs work with the same thermodynamic parameters for the nearest neighbor model as the Mfold program [43-46]. The programs are available by request.

## List of abbreviations

siRNAs – small interfering RNAs

miRNAs – micro RNAs

RISC – RNA-induced silencing complexes

RNAi – RNA interference

NN – neural network

## Authors' contributions

S.A. – performed computer analysis and drafted the ms

A.N. – performed computer analysis and drafted the ms

A.Y. – performed computer analysis and prepared the ms

## Note

^1 ^The training set cross-validation *R*^2 ^values are all averages over 50 random non-overlapping cross-validation splits. The variation in *R*^2 ^from one split to another is noticeable. The standard deviation of *R*^2 ^for a single split is 0.0022, 0.0063, and 0.0058 for the 4-parameter linear regression, 18-parameter neural network, and 4-parameter neural network, respectively. This large split-to-split *R*^2 ^variability reflects the variability (experimental error and differences in methods) in our training database.

## Supplementary Material

Additional File 1 FigureS1 Click here for file

Additional File 2 FigureS2 Click here for file

Additional File 3 FigureS3 Click here for file

Additional File 4 TableS1A Click here for file

Additional File 5 TableS1B Click here for file

Additional File 6 TableS2 Click here for file

Additional File 7 TableS3 Click here for file
