# Supplementary material for: Computational models with thermodynamic and composition features improve siRNA design
Source: BMC Bioinformatics. 2006 Feb 12;7:65. doi: 10.1186/1471-2105-7-65 (PMC1431570; doi:10.1186/1471-2105-7-65)
Supplement: Additional File 1 — FigureS1 [file 1471-2105-7-65-S1.doc]

**Figure 1.** Histograms of distributions for siRNA (A-F) and miRNA (E-F) features:

A) content index for preferred dinucleotides,

B) content index for avoided dinucleotides,

C) preferred position-dependent consensus index,

D) avoided position-dependent consensus index,

E) free energy differences between 5' and 3' terminal nucleotides duplexes,

F) free energy (ΔG) of sense-antisense duplexes.
