# Supplementary material for: Computational models with thermodynamic and composition features improve siRNA design
Source: BMC Bioinformatics. 2006 Feb 12;7:65. doi: 10.1186/1471-2105-7-65 (PMC1431570; doi:10.1186/1471-2105-7-65)
Supplement: Additional File 2 — FigureS2 [file 1471-2105-7-65-S2.doc]

**Supplementary Figure 2.** Overall block scheme illustrating siRNA feature selection approach.
