# Supplementary material for: Computational models with thermodynamic and composition features improve siRNA design
Source: BMC Bioinformatics. 2006 Feb 12;7:65. doi: 10.1186/1471-2105-7-65 (PMC1431570; doi:10.1186/1471-2105-7-65)
Supplement: Additional File 3 — FigureS3 [file 1471-2105-7-65-S3.doc]

**Supplementary Figure 3.** An example demonstrating how the summarized position-dependent consensus and avoided dinucleotide content index are calculated from siRNA sequence **U_1_G_2_U_3_C_4_U_5_G_6_A_7_G_8_C_9_A_10_A_11_A_12_U_13_A_14_A_15_G_16_C_17_G_18_C_19_**.

**A**

**Preferred position-dependent consensus index** =
 = δ(**A_1_**) + δ(**A_7_**) + δ(**A_10_**) + δ(**A_14_**) + δ(**C_17_**) + δ(**C_18_**) + δ(**C_19_**) + δ(**U_1_**) + δ(**U_2_**)
+ δ(**U_3_**) + δ(**U_5_**) + δ(**U_7_**) + δ(**U_13_**) + δ(**U_14_**)

**Avoided position-dependent consensus index** =
= δ(**A_5_**) + δ(**A_17_**) + δ(**A_11_**) + δ(**A_19_**) + δ(**C_1_**) + δ(**C_7_**) + δ(**C_14_**) + δ(**G_1_**) + δ(**G_2_**)
+ δ(**G_3_**) + δ(**G_6_**) + δ(**G_13_**) + δ(**G_14_**) + δ(**G_16_**) + δ(**U_19_**),

where δ(**N_i_**) = 1, if sequence has nucleotide **N** in *i*-th position; 0 - otherwise.

Preferred position-dependent consensus index =
= 0 + 1 + 1 + 1 + 1 + 0 + 1 + 1 + 0 + 1 + 1 + 0 + 1 + 0 = **9**

Avoided position-dependent consensus index =
= 0 + 0 + 1 + 0 + 0 + 0 + 0 + 0 + 1 + 0 + 1 + 0 + 0 + 1 + 0 = **4**

**B**

**Content index for avoided dinucleotides** = **N_CA_** + **N_GC_** + **N_GG_**, - sum of dinucleotides CA, GC and GG occurrences in siRNA.

Content index for avoided dinucleotides =
= 1 + 3 + 0 = **4**

**C**

**Normalization of input parameters**.

Normalization of parameters in preparation for input into neural network was performed as follows:

(*P^k^_i_* – *P^k^*_min_)

*P^k^_i_*_,normalized_ = -------------------,

(*P^k^*_max_ – *P^k^*_min_)

where *P^k^_i_* - analyzed *k*-th parameter for *i*-th siRNA, *P^k^*_max_ = max*_i_*(*P^k^_i_*), *P^k^*_min_ = min*_i_*(*P^k^_i_*) – corresponding maximum and minimum of *k*-th parameter for whole siRNA set.
