# Supplementary material for: Computational models with thermodynamic and composition features improve siRNA design
Source: BMC Bioinformatics. 2006 Feb 12;7:65. doi: 10.1186/1471-2105-7-65 (PMC1431570; doi:10.1186/1471-2105-7-65)
Supplement: Additional File 6 — TableS2 [file 1471-2105-7-65-S6.doc]

**Table 2. Correlation between the 18 parameters described siRNA features within efficient and inefficient siRNAs.**

|  |  |  |  |  |  |  |  |  |  |  |  |  |  |  |  |  |  |  |  |
| --- | --- | --- | --- | --- | --- | --- | --- | --- | --- | --- | --- | --- | --- | --- | --- | --- | --- | --- | --- |
|  |  |  | Consensus | | | ΔG | | | | ΔG of position | | | | | | Dinucleotide | |  |  |
|  | G | U | + | - | Σ | inter | intra | duplex | target | 1 | 2 | 6 | 13 | 14 | 18 | pref. | avoid. | Diff | Hybrid |
|  |  |  |  |  |  |  |  |  |  |  |  |  |  |  |  |  |  |  |  |
|  |  |  |  |  |  |  |  |  |  |  |  |  |  |  |  |  |  |  |  |
| G | 1 | -0.40 | 0.40 | -0.33 | -0.42 | -0.51 | -0.48 | -0.53 | -0.15 | -0.21 | -0.11 | -0.17 | -0.16 | -0.18 | -0.23 | 0.47 | -0.52 | -0.01 | -0.02 |
| U | -0.40 | 1 | -0.37 | 0.38 | 0.43 | 0.21 | 0.27 | 0.59 | 0.39 | 0.21 | 0.15 | 0.20 | 0.16 | 0.17 | 0.25 | -0.64 | 0.83 | 0.04 | 0.05 |
| Cons+ | 0.40 | -0.37 | 1 | -0.48 | -0.87 | -0.24 | -0.24 | -0.31 | -0.14 | -0.30 | -0.08 | -0.37 | -0.37 | -0.21 | 0.12 | 0.37 | -0.40 | 0.31 | 0.00 |
| Cons- | -0.33 | 0.38 | -0.48 | 1 | 0.85 | 0.21 | 0.21 | 0.29 | 0.17 | 0.34 | 0.19 | 0.19 | 0.37 | 0.23 | -0.15 | -0.33 | 0.37 | -0.36 | 0.05 |
| ConsΣ | -0.42 | 0.43 | -0.87 | 0.85 | 1 | 0.26 | 0.26 | 0.35 | 0.18 | 0.37 | 0.16 | 0.33 | 0.43 | 0.25 | -0.15 | -0.41 | 0.45 | -0.39 | 0.03 |
| ΔG inter | -0.51 | 0.21 | -0.24 | 0.21 | 0.26 | 1 | 0.67 | 0.47 | 0.15 | 0.19 | 0.21 | 0.18 | 0.13 | 0.19 | 0.09 | -0.40 | 0.24 | -0.07 | -0.03 |
| ΔG intra | -0.48 | 0.27 | -0.24 | 0.21 | 0.26 | 0.67 | 1 | 0.50 | 0.15 | 0.22 | 0.22 | 0.16 | 0.16 | 0.21 | 0.11 | -0.42 | 0.25 | -0.08 | -0.08 |
| ΔG duplex | -0.53 | 0.59 | -0.31 | 0.29 | 0.35 | 0.47 | 0.50 | 1 | 0.51 | 0.35 | 0.35 | 0.33 | 0.24 | 0.30 | 0.32 | -0.61 | 0.45 | -0.02 | -0.09 |
| ΔG target | -0.15 | 0.39 | -0.14 | 0.17 | 0.18 | 0.15 | 0.15 | 0.51 | 1 | 0.18 | 0.14 | 0.17 | 0.13 | 0.20 | 0.17 | -0.37 | 0.24 | 0.00 | -0.03 |
| Pos1 | -0.21 | 0.21 | -0.30 | 0.34 | 0.37 | 0.19 | 0.22 | 0.35 | 0.18 | 1 | 0.41 | 0.02 | 0.03 | 0.05 | 0.08 | -0.25 | 0.16 | -0.67 | -0.07 |
| Pos2 | -0.11 | 0.15 | -0.08 | 0.19 | 0.16 | 0.21 | 0.22 | 0.35 | 0.14 | 0.41 | 1 | 0.03 | -0.04 | 0.08 | 0.01 | -0.22 | 0.11 | -0.29 | -0.01 |
| Pos6 | -0.17 | 0.20 | -0.37 | 0.19 | 0.33 | 0.18 | 0.16 | 0.33 | 0.17 | 0.02 | 0.03 | 1 | -0.02 | -0.02 | 0.05 | -0.18 | 0.18 | 0.02 | 0.01 |
| Pos13 | -0.16 | 0.16 | -0.37 | 0.37 | 0.43 | 0.13 | 0.16 | 0.24 | 0.13 | 0.03 | -0.04 | -0.02 | 1 | 0.37 | 0.01 | -0.19 | 0.15 | -0.01 | -0.06 |
| Pos14 | -0.18 | 0.17 | -0.21 | 0.23 | 0.25 | 0.19 | 0.21 | 0.30 | 0.20 | 0.05 | 0.08 | -0.02 | 0.37 | 1 | -0.01 | -0.27 | 0.13 | -0.05 | -0.09 |
| Pos18 | -0.23 | 0.25 | 0.12 | -0.15 | -0.15 | 0.09 | 0.11 | 0.32 | 0.17 | 0.08 | 0.01 | 0.05 | 0.01 | -0.01 | 1 | -0.16 | 0.14 | 0.68 | -0.03 |
| Dinucl pref. | 0.47 | -0.64 | 0.37 | -0.33 | -0.41 | -0.40 | -0.42 | -0.61 | -0.37 | -0.25 | -0.22 | -0.18 | -0.19 | -0.27 | -0.16 | 1 | -0.50 | 0.06 | 0.00 |
| Dinucl avoid. | -0.52 | 0.83 | -0.40 | 0.37 | 0.45 | 0.24 | 0.25 | 0.45 | 0.24 | 0.16 | 0.11 | 0.18 | 0.15 | 0.13 | 0.14 | -0.50 | 1 | -0.01 | 0.11 |
| Diff | -0.01 | 0.04 | 0.31 | -0.36 | -0.39 | -0.07 | -0.08 | -0.02 | 0.00 | -0.67 | -0.29 | 0.02 | -0.01 | -0.05 | 0.68 | 0.06 | -0.01 | 1 | 0.03 |
| Hybrid | -0.02 | 0.05 | 0.00 | 0.05 | 0.03 | -0.03 | -0.08 | -0.09 | -0.03 | -0.07 | -0.01 | 0.01 | -0.06 | -0.09 | -0.03 | 0.00 | 0.11 | 0.03 | 1 |
|  |  |  |  |  |  |  |  |  |  |  |  |  |  |  |  |  |  |  |  |
